# Spectrum and Pattern of Movement Disorders in Patients with Sporadic Creutzfeldt-Jakob Disease

**DOI:** 10.5334/tohm.753

**Published:** 2023-05-03

**Authors:** Sandeep Gurram, Vikram V. Holla, Praveen Sharma, Nitish Kamble, Jitender Saini, Manjunath Netravathi, Ravi Yadav, Pramod Kumar Pal

**Affiliations:** 1 Department of Neurology, National Institute of Mental Health & Neurosciences, Hosur Road, Bengaluru-560029, Karnataka, India; 2 Department of Neuroimaging and Interventional Radiology, National Institute of Mental Health & Neurosciences, Hosur Road, Bengaluru-560029, Karnataka, India

**Keywords:** Movement disorders, Creutzfeldt-Jakob disease, Parkinsonism, Ataxia, Myoclonus

## Abstract

**Background:**

Creutzfeldt-Jakob disease (CJD) is a rare neuro degenerative disease that is mainly characterized by rapidly progressive dementia along with a varying combination of myoclonus, visual, cerebellar, pyramidal/extrapyramidal and akinetic mutism. Several movement disorders phenomenologies can occurs either at onset, as presenting symptom or during the course of illness. Present study aims to characterize the clinical, radiological features and the outcome of patients with CJD with movement disorders as the forthcoming manifestation.

**Methods:**

Chart review of patients with CJD with movement disorders. Demographic, clinical and radiological details of the patients were reviewed.

**Results:**

25 patients (13 males) of sCJD with median age at presentation of 58 years and median duration of illness of 5 months were included in the study. According to revised CDC diagnostic criteria 1 patient was classified as definite sCJD, 20 as probable and 2 as possible CJD. Myoclonus, ataxia and parkinsonism were the most common movement disorder and chorea was the least common. Magnetic resonance imaging of brain was performed in all and basal ganglia abnormality and cortical ribboning was seen in more than two-third of cases. Electroencephalographic abnormality was noted in 21 patients with triphasic waves and periodic sharp waves seen in 7 and 6 patients respectively. Cerebrospinal fluid 14-3-3 assay was abnormal in 2 out of 4 patients. Atypical presentations were noted in the form of ataxic presentation, CBS like presentation and choreiform presentation.

**Conclusion:**

Myoclonus, ataxia and parkinsonism are the most frequent movement disorders phenomenology observed in patients with sCJD.

## Introduction

Creutzfeldt-Jakob disease (CJD) is a rare neuro degenerative disease that is mainly characterized by rapidly progressive dementia, myoclonus, ataxia, visual disturbances, extrapyramidal and pyramidal involvement, as well as akinetic mutism [1,2,3]. It is subdivided into 4 types namely sporadic, familial, iatrogenic and variant CJD. It has various types of clinical presentation including classical, ataxic, pure cognitive, psychiatric, visual, thalamic, stroke like and CBS like presentations [4]. CDC has devised a new set of diagnostic criteria for CJD in 2017 that includes the MRI findings and RTQuIC in the diagnosis when compared to World Health Organization (WHO) 1998 criteria [5,6].

Several movement disorders including myoclonus, dystonia, choreoathetosis, tremor, hemiballismus, ‘ill-defined complex’ and atypical parkinsonian syndromes, i.e., corticobasal syndrome (CBS) and supranuclear palsy (PSP), has been described in a significant number of patients with a sporadic, familial or new variant of CJD (v-CJD) [7,8,9]. The frequency of movement disorders increases with disease duration, but they sometimes occur at an early stage [2] and have been described as initial and/or isolated manifestations of the illness. The most common movement disorder seen in patients with CJD is ataxia followed by parkinsonism and myoclonus [10,11,12]. CJD can also present with various other movement disorders such as chorea, dystonia and tremor.

There is a paucity of data on the pattern and prevalence of movement disorders associated with CJD in India. Even the studies done outside India have variability in the pattern of movement disorders associated with CJD. Hence there is a need to identify the common patterns of movement disorders associated with CJD and their prevalence that can help in early disease identification as well as predicting the stage of the disease.

## Methodology

It is a retrospective, single-centre study done at the National Institute of Mental Health and Neurosciences (NIMHANS). All evaluated patients of CJD from the database of the movement disorder team at NIMHANS from January 2013 to April 2022 were included. Demographic details, such as age, gender, occupation, duration of illness and geographical location; clinical features and available investigation findings including MRI brain, electroencephalography and autopsy data were collected from the patient’s case file. The patients identified were segregated into sporadic, familial, variant, or iatrogenic subtypes based on suspected aetiology and classified according to the 2017 CDC diagnostic criteria into definite, probable, or possible CJD [5,6]. The various movement disorders were identified as per documented clinical findings available in the case files after clinical evaluation and in addition, patient videos where available were also reviewed. Institute Ethics Committee approval was obtained for the retrospective analysis of the data and informed consent was obtained from the patients before video recording for recording and publication.

### Statistical Analysis

Data were expressed using descriptive statistics, where median and range was used for continuous variables, and frequencies and percentages were used for categorical variables.

## Results

**Figure 1:**
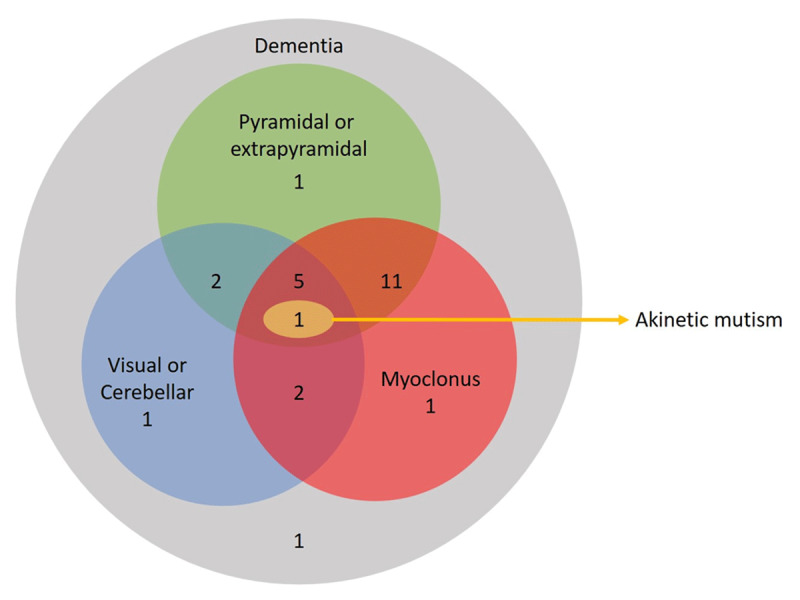
**Clinical domains.** A Venn diagram demonstrating the combination of various clinical domains observed in patients with sCJD at the time of presentation.

A total of 25 patients (13 males) were included in the study, all of which were sporadic CJD. The median age at the presentation of the cohort was 58 years (range: 40–76) with a median duration of illness of 5 months (range: 1 – 12). One patient was diagnosed with definite CJD, 18 patients were probable CJD and 2 patients were possible CJD according to the CDC diagnostic criteria. Four patients could not be classified according to the CDC diagnostic criteria at the time of presentation owing to only one clinical domain involvement apart from dementia at the time of presentation but otherwise had clinical, EEG and imaging characteristics consistent with the diagnosis of CJD with the alternative diagnosis ruled out by relevant diagnostic investigations (autoimmune, paraneoplastic panel, anti-TPO, and CSF analysis were normal). Two of these 4 patients developed new clinical signs in the follow-up consistent with probable sCJD resulting in a total of 20 probable sCJD. Autopsy was performed in only one patient who was confirmed to have definite sCJD. All patients had dementia, 21 patients had myoclonus, 21 patients had pyramidal or extrapyramidal signs, 11 patients had visual or cerebellar signs and 1 patient had akinetic mutism in various combinations (Figure 1).

### The spectrum of movement disorders

**Figure 2:**
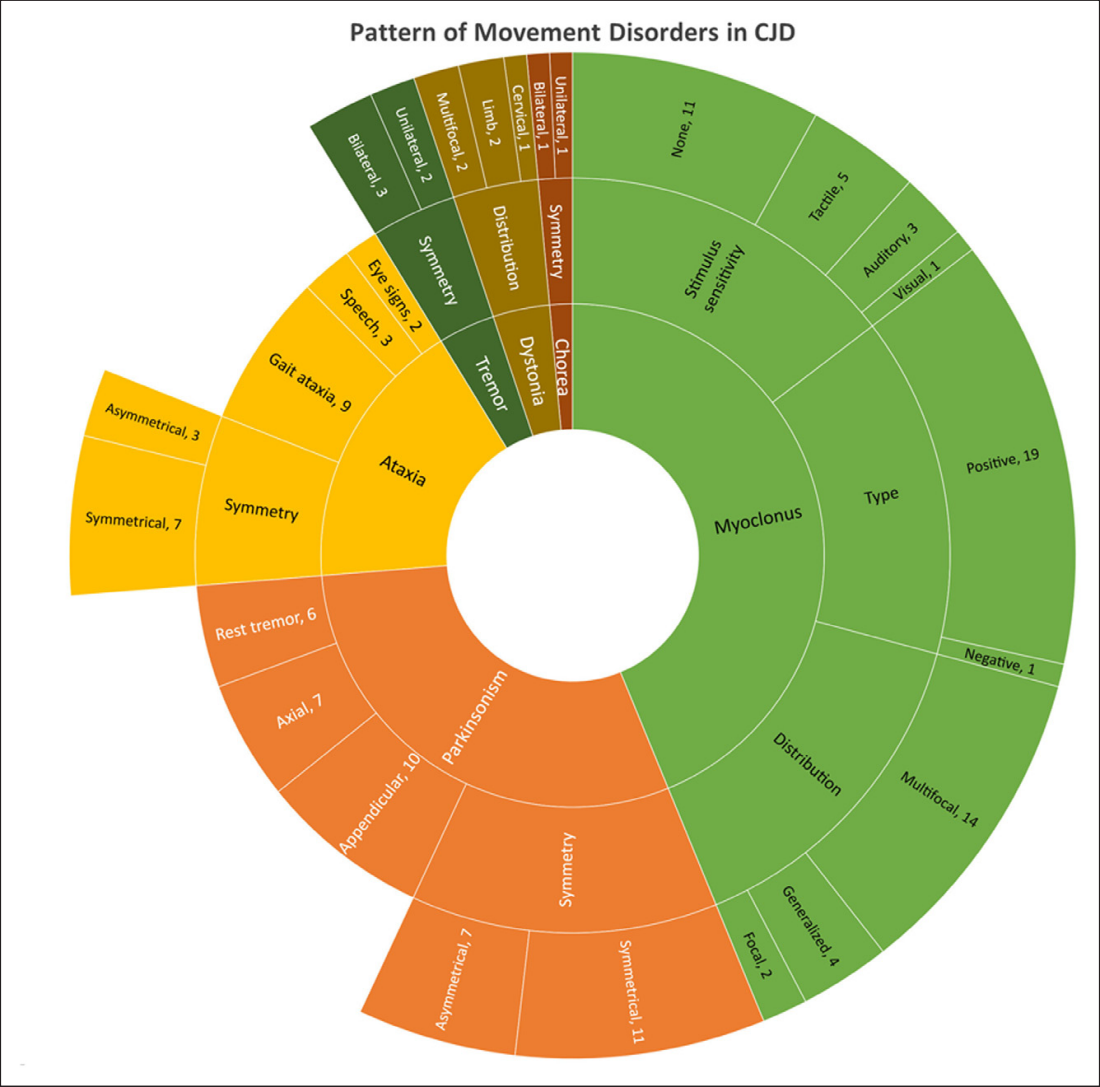
**Pattern of movement disorders.** Sunburst diagram showing the pattern of various movement disorders phenomenology in patients with sCJD at the time of presentation.

Movement disorders were seen in 24 out of 25 patients (Table 1, Figure 2) at the time of presentation. The most common movement disorder was myoclonus (20), followed by parkinsonism (18), ataxia (10), dystonia (5), tremor (5) and choreoathetosis (2). Multiple movement disorders (>1) were seen in 20 patients. Multifocal myoclonus was seen in 14 patients, generalized myoclonus in 4 patients and focal limb myoclonus in 2 patients. Isolated spontaneous myoclonus was seen in 11 patients and stimulus-sensitive myoclonus was noted in 9 patients. The somatosensory stimulus was the most common stimulus seen in 5 patients, auditory stimulus in 3 patients and visual stimulus in 1 patient (Video 1). One patient had negative myoclonus with asterixis. Asymmetrical parkinsonism was seen in 7 patients and the remaining 11 patients had symmetrical parkinsonism. Jerk intermittent rest tremor was seen in 6 patients but none had a classical pill rolling component. Axial predominant rigidity was seen in 7 patients and 10 patients had predominant appendicular rigidity. One patient had no rigidity but only bradykinesia and tremor. A patient with parkinsonism had asymmetrical rigidity, bradykinesia, dystonia and stimulus-sensitive myoclonus mimicking CBS (Video 2) later proven to sCJD on autopsy. Gait ataxia was the predominant clinical manifestation in 9 out of 10 patients with cerebellar signs (Video 3). Isolated limb ataxia was seen in only 1 patient.

**Table 1:** Demographic, clinical and investigation findings of the cohort of CJD (n = 25 patients).


VARIABLE	N (%)

Age at presentation (median, range)	58 years (40–76)

Duration of illness (median, range)	5 months (1–12)

Gender (Male: Female)	13:12

***Clinical features***	

Altered sensorium	3 (12%)

Seizures	4 (16%)

Focal Neurological Deficits	6 (24%)

(a) Visual disturbances	2 (8%)

(b) Aphasia	2 (8%)

(c) Hemiparesis	1 (4%)

(d) Others	1 (4%)

Dementia	25 (100%)

Movement disorders	24 (96%)

(a) Myoclonus	20 (80%)

(b) Parkinsonism	18 (72%)

(c) Ataxia	10 (40%)

(d) Dystonia	5 (20%)

(e) Tremor	5 (20%)

(f) Chorea	2 (8%)

Multiple movement disorders (>1)	20 (80%)

***Investigations***	

Magnetic resonance imaging of the Brain	

(a) Bilateral basal ganglia T2/FLAIR hyperintensities	20 (80%)

(b) Cortical ribbon sign	16 (64%)

(c) Double hockey stick sign	8 (32%)

(d) Diffusion restriction (DWI available in 20 patients)	20 (80%)

Cerebrospinal fluid (CSF) analysis	

(a) CSF Pleocytosis	2 (8%)

(b) Elevated CSF protein	10 (40%)

Electroencephalographic findings	

(a) Diffuse slowing	17 (68%)

(b) Triphasic waves	6 (24%)

(c) Periodic sharp wave complexes	7 (28%)

**Diagnosis of CJD at presentation according to CDC criteria**	

(a) Definite	1 (4%)

(b) Probable	20 (80%)

(c) Possible	2 (8%)

(d) Not classifiable	2 (8%)


CJD: Creutzfeldt-Jakob disease; CDC: Centre for disease control; DWI: Diffusion-weighted imaging.

**Video 1:** **A patient of sCJD with generalized myoclonus.** A patient with probable sCJD having spontaneous generalized myoclonus along with visual sensitivity. EEG showed periodic sharp wave discharges.

**Video 2:** **A patient with an ataxic presentation of sCJD.** A patient with probable sCJD with ataxic presentation having reduced blink rate and facial expression, cerebellar dysarthria, appendicular incoordination and gait ataxia.

**Video 3:** **A patient with a CBS-like presentation of sCJD.** A patient with definite sCJD having CBS-like presentation with right side predominant bradykinesia, rigidity, dystonia, apraxia and stimulus-sensitive myoclonus. The patient rapidly progressed over 1 month to a bedridden state and later succumbed to the illness.

Multifocal dystonia was seen in 2 patients and only focal limb dystonia was seen in 2 patients. One patient had cervical dystonia alone. Choreoathetosis was seen in 2 patients, unilateral in one and bilateral in the other (Video 4). Postural tremors were seen in 5 patients, but all of them were associated with parkinsonism and one of them had additional rest tremor as well. Two patients had unilateral and 3 patients had bilateral tremors.

**Video 4:** **A patient of sCJD with chorea.** A patient with probable sCJD presenting with subacute generalized chorea at the onset.

### Investigations

MRI brain and EEG were performed in all patients. MRI brain was abnormal in all and showed bilateral basal ganglia T2/FLAIR hyperintensities in 20, cortical ribbon sign in 16 and double hockey stick sign in 8 patients. In the 8 patients with hockey stick sign, there was decreasing anteroposterior gradient with the hyperintensity more in the caudate and putamina compared to the thalamic hyperintensity unlike what is seen classically in vCJD (thalamic hyperintensity more compared to caudate-putamen) [13]. Diffusion-weighted imaging was available in 20 patients and showed diffusion restriction of the involved regions in all. EEG was abnormal in 21 patients showing diffuse slowing in 17, triphasic waves in 6 and periodic sharp wave complexes in 7 patients. Cerebrospinal fluid analysis was performed in 23 patients out of which 2 patients had CSF pleocytosis (median: 2.5 cells/µl, range: 0–19 cells/µl) and 10 patients had elevated CSF protein (median: 53, range 5.7–92 mg/dl). CSF 14-3-3 assay was performed in 4 patients and 2 were positive whereas RT-QuIC was not done in any of the patients. In addition to these, serum autoimmune encephalitis (NMDA, CASPR-2, LGI-1, GABA-B, AMPA1, AMPA2), paraneoplastic (anti-GAD, Ma1/Ma2, CRMP-5, amphiphysin, Yo, PCA2, Hu, Ri) antibody panels and anti- were assessed in 21 and 19 patients respectively and all were negative.

## Discussion

In this retrospective case series, we report the spectrum of movement disorder phenomenology in 25 patients of sCJD. Variant, familial and iatrogenic CJD cases were not found in our series and all but two cases eventually satisfied at least possible sCJD criteria according to CDC criteria. This could probably be due to numerous variants of CJD described like pure ataxic variant, visual variant, pure cognitive variant, thalamic variant, behavioural variant or stroke-like variant that won’t fit into the criteria for sCJD [10]. These patients later may or may not evolve into the classical pattern of sCJD. Two of the 4 patients had an ataxic variant and stroke-like presentation and the other 2 patients had a mean duration of illness of around 4 months after which they were lost for follow-up. The majority of the patients in our cohort had less than 6 months duration of illness at the time of presentation.

Movement disorders are reported in almost all patients affected with CJD either at the onset or during the course of illness [11]. A wide spectrum of akinetic, hypokinetic to hyperkinetic phenomenologies can be observed. Ataxia and myoclonus are the most common phenomenologies observed followed by parkinsonism, tremor, dystonia and chorea. In a recent longitudinal cohort study of prion diseases [14], around 90% had gait disturbance, either ataxic or apraxic at presentation and around 70% had myoclonus. The increased tone was noted in 66% among which three-fourths were due to rigidity. Tremor and chorea were the least common movement disorders seen in less than 10% of cases [10,11,14]. Similar findings were noted in a systematic review of movement disorders in prion diseases where ataxia, myoclonus and rigidity were noted in around 40–60% of cases of sCJD and dystonia, tremor and chorea were the least common phenomenology seen in around 5% of cases. When compared to sCJD, ataxia and chorea are more frequent while myoclonus and rigidity are less frequent in vCJD [11,14]. Similar findings are observed in the current cohort as well with frequent occurrence of myoclonus and ataxia. However, parkinsonism was noted in around 75% of cases compared to around 10–40% according to previous studies [11,14].

Myoclonus is usually distal predominant and multifocal at onset and tends to become diffuse and generalized with prevalence reaching 100% with disease progression. It is often spontaneous but can be sensitive to auditory, visual and/or tactile sensations and they can persist during sleep [10]. In the study by Sequeira et al, spontaneous myoclonus was noted in 51% of patients, stimulus sensitivity in 45% and startle myoclonus in 43%. This was similar to our study with 50% having spontaneous myoclonus and the rest having either tactile, visual and/or startle sensitivity. Although less common, negative myoclonus has been described in patients with CJD and was seen in one patient of our cohort [15,16]. Both positive and negative myoclonus in CJD have a cortical origin and is substantiated by radiological and electrophysiological evidence. They are associated with periodic sharp-wave discharges on EEG. Despite the EEG changes, seizures are observed in less than 1% at presentation and in around 10–25% of cases with disease progression [7,10,17].

Ataxia is a common manifestation at onset and can precede the onset of significant cognitive impairment otherwise known as the Brownell-Oppenheimer variant of sCJD [18]. Gait ataxia is more frequent compared to limb ataxia and is seen in around two-thirds of the cases. Although gait ataxia is common in all types of CJD, it is more prevalent in vCJD compared to other types. Whereas, limb ataxia is less prevalent in Gerstmann-Strausssler-Sceinker (GSS) disease compared to other prionopathies. More than 80% of patients with ataxia in our cohort had gait ataxia and it was symmetrical in close to three-fourths of the patients. Ataxia can be either secondary to cerebellar involvement or due to the pathology involving cerebellar outflow tracts in the thalamus.

Patients with CJD can mimic presentations of various atypical parkinsonism types and vice-versa. Axial predominant rigidity seen in CJD along with early gait abnormality may mimic progressive supranuclear palsy and there are reports of both sCJD and familial CJD diagnosed as PSP in the early stage [10,19,20]. Oculomotor abnormalities such as gaze abnormalities have also been described in CJD further complicating the diagnosis [14]. Additionally, asymmetric rigidity with stimulus-sensitive myoclonus can mimic cortico-basal syndrome with the rare occurrence of alien limb phenomenon with CJD adding to the conundrum [21,22]. One case in our cohort had such a CBS-like presentation with asymmetrical rigidity, parkinsonism, dystonia and stimulus-sensitive myoclonus and later rapidly progressed and eventually succumbed to illness and was confirmed to have sCJD on autopsy. Frontotemporal dementia with parkinsonism and dementia with Lewy body degeneration are the other parkinsonism that can mimic CJD presentation and are sometimes difficult to differentiate [23].

Other movement disorders such as dystonia, tremor and chorea are less frequent and often does not exist alone. According to a systematic review, the prevalence of dystonia, tremor and chorea in sCJD is 9.1%, 4.9%, and 3.7% respectively [11]. Dystonia is usually distal and focal affecting the upper limb or neck. In a few cases, it can be cervical, segmental or also hemidystonia [24,25]. Tremors can be unilateral or bilateral, rest, kinetic or postural involving the upper limb predominantly. Rest tremor is uncommon and is often jerky and should look for superimposed dystonic tremor or spontaneous myoclonus mimicking tremor [10]. Chorea is one of the least common movement disorders seen in less than 5–10% of cases but it is disproportionately more common in vCJD. Rarely chorea as a presenting and debilitating symptom can occur in sCJD as seen in one of our cases.

The frequent occurrence of movement disorders phenomenology in CJD is reflected in imaging findings as well with basal ganglia and thalamus involvement in the majority of the patients. Cortical involvement can explain myoclonus, alien limb phenomenon and dystonia as well. When the clinical features are focal, lateralized or asymmetrical, contralateral predominant changes can be seen on imaging. [24,26,27]. Similar asymmetrical findings can be noted in EEG findings as well as correlating with lateralizing or asymmetrical clinical findings. In the case mimicking CBS from our cohort, the patient had right-side predominant clinical findings correlating with left-side predominant cortical and basal ganglia changes.

CSF analysis is important for both to rule out the alternate cause as well as for 14-3-3 and RT-QUIC assay which increases the sensitivity and specificity of the diagnostic criteria. RT-QuIC assay has better sensitivity and specificity compared to 14-3-3 which is reflected in the improved sensitivity without altering the specificity in the modified CHD diagnostic criteria [6,28]. Neuropathology studies corroborate the imaging findings with basal ganglia and thalamus being the most common non-cortical brain area affected. Myoclonus and the periodic slow wave discharges are postulated to be secondary to the loss of inhibitory neurons in the reticular thalamus. The various movement disorder phenomenologies that develop in CJD are possibly secondary to the vacuolation, neuronal loss, and astrocytosis in various circuits involving the cortical, striato-pallidal, thalamic, mesencephalic and cerebellar complex.

### Limitations

The retrospective case-file-based nature of the study is a major limitation. In addition, ours being a tertiary referral centre for movement disorders, there may be a bias leading to more cases of movement disorders. The sample size of the population is also too small for drawing large-scale conclusions, but still, it provides a good overview of the prevalence and pattern of movement disorders observed in a relatively rare disease in the Indian context which is currently lacking. Finally, autopsy could be performed in only one patient, CSF 14-3-3 in only 4 patients and due to non-availability of CSF RT-QuIC test, it could not be performed in any of the patients adding to the limitations.

## Conclusion

Movement disorders often in multiple combinations are frequently seen in patients with Creutzfeldt-Jakob disease. Myoclonus, ataxia and parkinsonism are the most frequent phenomenologies whereas dystonia, tremor and chorea are infrequent. CJD can present with uncommon presentations such as CBS mimic, ataxic or choreiform presentation and one needs to be aware of such presentations for prompt diagnosis.
